# Genome-Wide Identification of *14-3-3* gene family reveals their diverse responses to abiotic stress by interacting with StABI5 in Potato (*Solanum tuberosum L*.)

**DOI:** 10.3389/fpls.2022.1090571

**Published:** 2023-01-09

**Authors:** Qianqian Wang, Chongchong Yan, Yuying Fu, Yu Wang, Pengfei Jiang, Yinyuan Ding, Huajun Liao

**Affiliations:** ^1^ Key Laboratory of Horticultural Crop Germplasm innovation and Utilization, Institute of Horticulture, Anhui Academy of Agricultural Sciences, Hefei, China; ^2^ National Engineering Laboratory of Crop Stress Resistance, School of Life Sciences, Anhui Agricultural University, Hefei, China

**Keywords:** *14-3-3* gene family, abscisic acid, abiotic stress, potato, *StABI5*

## Abstract

The *14-3-3* genes are widely present in plants and participate in a wide range of cellular and physiological processes. In the current study, twelve *14-3-3s* were identified from potato genome. According to phylogenetic evolutionary analysis, potato *14-3-3s* were divided into ϵ and non-ϵ groups. Conserved motif and gene structure analysis displayed a distinct class-specific divergence between the ϵ group and non-ϵ group. Multiple sequence alignments and three-dimensional structure analysis of 14-3-3 proteins indicated all the members contained nine conservative antiparallel α-helices. The majority of *14-3-3s* had transcript accumulation in each detected potato tissue, implying their regulatory roles across all stages of potato growth and development. Numerous cis-acting elements related to plant hormones and abiotic stress response were identified in the promoter region of potato *14-3-3s*, and the transcription levels of these genes fluctuated to different degrees under exogenous ABA, salt and drought stress, indicating that potato *14-3-3s* may be involved in different hormone signaling pathways and abiotic stress responses. In addition, eight potato 14-3-3s were shown to interact with StABI5, which further demonstrated that potato 14-3-3s were involved in the ABA-dependent signaling pathway. This study provides a reference for the identification of the 14-3-3 gene family in other plants, and provides important clues for cloning potential candidates in response to abiotic stresses in potato.

## Introduction

The 14-3-3 proteins have been demonstrated to be ubiquitously present in eukaryotes and is recognized as one group of the most critical phospholipid binding proteins (Sehnke et al., 2000; Sehnke et al., 2002a; Paul et al., 2012). They were first identified from cow brain tissue by Moor and Perez, accounting for about 1% of all soluble proteins in brain tissue so that they are considered as brain specific for a long time (Moore and Perez, 1967)). Subsequent studies found that the 14-3-3 proteins present in various tissues of all multicellular eukaryotic organisms, and they were extremely conserved in evolution from unicellular eukaryotes to high plants and animals (Ferl, 1996; Sehnke et al., 2002a). The identified 14-3-3 proteins are relatively tiny acid proteins (25~32 kDa) consisting of nine typically antiparallel α-helices that result in forming homo/hetero dimers (Dong et al., 1995; Yang et al., 2006). It is intriguing that both of the 14-3-3 proteins in the dimer are capable of binding to two different target proteins or two binding sites of one protein respectively, which makes them function as scaffolding proteins or adaptor proteins to involved in various biological pathways, such as enhancing or inhibiting the catalytic activity of target proteins, regulation of nuclear transport and subcellular localization of target proteins, changing the binding ability of target proteins to other proteins and avoiding target protein degradation (Sehnke et al., 2002a; Aitken, 2006; Ryu et al., 2007; Latz et al., 2010; Paul et al., 2012; Cotelle et al., 2014). It is reported that 14-3-3 proteins bind to target protein through 3 types of phosphorylation modes, including R(S/Ar)(+)p(S/T)XP, RX(Ar)(+)p(S/T)XP and PSX1-2-COOH, of which P (S/T) represents phosphorylated serine/threonine, Ar represents aromatic amino acids, + represents basic amino acids, and X after p(S/T) represents any types of amino acids, but it is usually leucine, glutamic, alanine or methionine (Yaffe et al., 1997; Ganguly et al., 2005).

Compared with 14-3-3 proteins in animals, plant 14-3-3 proteins were found relatively late, and they were first identified from model plant *Arabidopsis thaliana* as well as *Spinacia oleracea*, *Oenothera hookeri* and *Hordeum vulgare* (Brandt et al., 1992; Hirsch et al., 1992; Lu et al., 1992). With the development of molecular biology techniques, an increasing number of 14-3-3 proteins have been identified in various plants, such as rice (8), tomato (12), common bean (18), banana (25), apple (18) and so on (Sehnke et al., 2002b; Chen et al., 2006; Xu and Shi, 2006; Li et al., 2021; Lyu et al., 2021; Shao et al., 2021). As a research hotspot, 14-3-3 proteins have received more and more attention from researchers in plant researches. Studies have shown that 14-3-3s can bind to the plant plasma membrane H+-ATPase enzymes involved in phosphorylation to enhance its activity (Wilson et al., 2016). Besides, plant 14-3-3 proteins are also able to be modified through conformation and combined with the signal proteins to regulate the physiological and biochemical reactions. For example, 14-3-3s can interact with signal proteins through phosphorylation, acetylation or ubiquitination to regulate starch synthesis metabolism and protein transport during maize grain development (Yu et al., 2016). In addition, a plenty of previous studies have demonstrated that plant 14-3-3s are also involved in other biological processes, including primary metabolism, signal transduction, ion transport, transcriptional regulation, biotic and abiotic stress response (Ganguly et al., 2005; Schoonheim et al., 2007; Denison et al., 2011; Chen et al., 2017; Huang et al., 2022).

In the long-term evolution, plants have formed a set of complex mechanisms to respond to various abiotic stresses, such as drought, high temperature, cold and salt stress (Zhu, 2016; Gong et al., 2020). As extremely conserved regulatory proteins, 14-3-3s are at the intersection of the plant’s response to abiotic stress signal network and respond to a variety of stresses (Fu et al., 2000). For instance, the expression of four rice 14-3-3 genes, OsGF14b, OsGF14c, OsGF14e and OsGF14f are induced by drought, low temperature and salt stress, and overexpression of OsGF14c in rice enhances the drought tolerance of transgenic seedlings (Chen et al., 2006). Additional analysis shows that this process depends on the regulation of OsCDPK1 by OsGF14c. In Arabidopsis, two 14-3-3 proteins λ and κ have been confirmed to negatively regulate salt tolerance by interacting with the salt-sensitive protein SOS2 to inhibit its kinase activity (Zhou et al., 2014). Overexpressing of GsGF14o in Arabidopsis significantly reduce drought tolerance during seed germination and seedling growth. Further analysis showed that transgenic plants exhibited lower stomata opening, incomplete root hair development, reduced transpiration rate and water intake, which indicates that GsGF14o plays a negative regulatory role in drought tolerance (Sun et al., 2013).

Potato (*Solanum tuberosum* L.) is used as crop grain, vegetable or industrial raw material, which is grown all over the world and plays an essential role in solving hunger and ensuring food (Halterman et al., 2016). As the characteristics of comprehensive nutrition and wide adaptability, in 2015, potato was listed as the fourth staple food crop after maize, wheat and rice in China. Potato cultivation is exposed to the external environment, hence, it is frequently affected by various stress factors during the whole growth cycle, such as abiotic stress (eg, drought, salinity, freezing damage, flood, and elevated temperature) and biotic stress (virus, fungal pathogens, bacterial pathogens). These environmental stress factors result in reduced potato yield and quality. Plants can transmit identified stress signals through signal transduction pathways, thereby activating the expression of stress-related genes that regulate the plant’s tolerance to the corresponding stress (Gong et al., 2020). Therefore, it is increasingly important and urgent for potato production and molecular breeding to explore stress response genes and elucidate their molecular mechanisms. The 14-3-3 proteins have been confirmed to play momentous roles in various stress responses in kinds of plant (Roberts et al., 2002). Although some individual *14-3-3s* were reported in potato (Żuk et al., 2003; Aksamit et al., 2005), the specific information and molecular mechanisms of potato14-3-3 gene family members were still poorly understood. In this study, we performed a comprehensive analysis of potato 14-3-3 gene family through bioinformatics methods at genome-wide level. A total of 12 potato *14-3-3s* were identified, then we analyzed their phylogenic relationships, chromosomal locations, gene structures, cis-elements, conserved motifs, and subcellular locations, as well as characterized their expression in responses to ABA, drought and salt stress. Furthermore, eight 14-3-3s were shown to be able to interact with StABI5, a key member in ABA-dependent pathways. These results provide a foundation for further functional studies of the *14-3-3s* in potato.

## Materials and methods

### Plant materials and stress treatments

The sequenced doubled monoploid potato plants DM1-3 (*S.phureja*) were used in this study. Potato seedlings *in vitro* were cultured in an artificial climate chamber with a photoperiod of 16 h light/8 h dark at temperature 24 ± 2 °C and 55% relative humidity. The stress treatment method for potato seedlings was according to previously described protocols and made some modifications (Charfeddine et al., 2015). Four-weeks-old seedlings were sprayed with 100 μM ABA (abscisic acid) or submitted to 15% polyethylene glycol 6000 (PEG 6000) and 100 mM sodium chloride (NaCl), which simulated the drought and salt stresses, respectively. Then the treated materials were harvested at the indicated time points, 0, 1, 6 and 12 h, and immediately frozen in liquid nitrogen for further analysis. Seedlings grown in normal condition without treatment were served as control. All the samples were conducted three biological replicates.

### Identification of 14-3-3s in potato and other plants

The potato genome database version 4.03 of the doubled monoploid S. tuberosum Phureja DM1-3 was downloaded from Spud DB Potato Genomics Resources website (http://spuddb.uga.edu/), then two approaches were used to identify potato *14-3-3s*. Firstly, we constructed a potato protein local database and searched against it using the query of 14-3-3 proteins Hidden Markov Model (HMM) sequence (PF00244.20) through BLASTP program (E-value set as 0.001) (Tang et al., 2016). Secondly, we downloaded the 14-3-3 family protein sequences of *Arabidopsis thaliana*, *Solanum lycopersicum*, *Zea mays* and *Brachypodium distachyon* from Phytozome v13 (https://phytozome-next.jgi.doe.gov/) according to reported protein ID (**Table S2**) (Cao et al., 2016). All the downloaded sequences were employed to search against the potato protein database to identify all potential 14-3-3s. The Pfam (http://pfam.xfam.org/) and SMART (http://smart.embl-heidelberg.de/) databases were used to further check the candidates on the basis of presence of conserved 14-3-3 domain (Schultz et al., 1998; Finn et al., 2013). Finally, repeated and incomplete sequences were manually removed. The ExPASy database (http://www.expasy.org/tools/) was employed to identify isoelectric points (PI) and molecular weights (MW) of potato 14-3-3 proteins.

### Characterization of 14-3-3s sequences, phylogenetic tree construction and cis-element analysis

The identified potato full-length 14-3-3 protein sequences were employed to perform multiple sequence alignment with an inner Muscle program of MEGA version 7.0 software (http://www.megasoftware.net/ ) (Kumar et al., 2016), then an un-root phylogenetic tree was constructed through maximum likelihood method (ML)with 1000 bootstrap replicates. To investigate the phylogenetic relationships among different plants, the same method was used to generate the other phylogenetic tree with 14-3-3 proteins from potato, *Arabidopsis*, tomato, maize and *Brachypodium*. The online Gene Structure Display Server 2.0 program (GSDS) (http://gsds.cbi.pku.edu.cn/ ) was applied to determine the exon–intron structure of potato *14-3-3s* based on their CDS sequences and corresponding DNA sequences (Hu et al., 2014). The Multiple Expectation maximization for Motif Elicitation (MEME) program (http://meme-suite.org/tools/meme ) was employed to analyze distribution of conserved motifs in potato 14-3-3s with parameters of optimum width ranges from 6 to 200, maximum number of motifs being 15 and any number of repetitions (Bailey et al., 2009). Then the identified motif sequences were further annotated using Pfam and SMART databases. Based on the location information of potato *14-3-3s* in the genome database, the physical chromosome location image of each gene was generated using MapInspect software (https://mapinspect.software.informer.com/ ). The DNAMAN version 6.0 software was adopted to construct the multiple alignments of potato 14-3-3s, and three-dimensional structure of each protein was predicted using the online Phyre^2^ program with normal mode (http://www.sbg.bio.ic.ac.uk/phyre2/html/page.cgi?id=index ) (Kelley et al., 2015). The upstream 1.5 kb genomic sequence from the start codon of potato 14-3-3 genes were derived, which was further submitted to PlantCARE database (http://bioinformatics.psb.ugent.be/webtools/plantcare/html/ ) to detect the putative cis-elements in promoter region of each gene (Lescot et al., 2002).

### Expression pattern analysis using transcriptome data

Genome-wide transcriptome data of potato DM1-3 (*S.phureja*) was downloaded from Spud DB Potato Genomics Resources website (http://spuddb.uga.edu/ ) (Massa et al., 2011). The fragments per kilobase of exon model per million mapped reads (FPKM) values of each *14-3-3s* were retrieved. Referring to previous study (Kumar et al., 2013; Wang et al., 2015), the FPKM data was further processed. For the spatial and temporal expression, FPKM valves were transformed by taking log_2_ (FPKM +1), thereafter, the processed data was loaded into R software to perform cluster analysis and generate a heatmap.

### RNA extraction and quantitative real-time PCR analysis

The Trizol reagent (Invitrogen, USA) was adopted for total RNA isolation of all collected samples referring to manufacturer’s protocol. 2 μg of extracted RNA was measured for reverse transcription into cDNA using PrimeScript™ RT reagent Kit with gDNA Eraser (TaKaRa). Potato 14-3-3s specific primers for this experiment were designed through Primer Express 3.0 software (Applied Biosystems), and the specificity of all primers were further verified in NCBI database with Primer Blast program (**Table S3**). The *EF1α* (Elongation factor 1*α*) gene was served as an internal control (Charfeddine et al., 2015). The qPCR was performed on an ABI Quant studio 3 Real-Time system (Applied Biosystems) using SYBR^®^ Green Realtime PCR Master Mix (TOYOBO, Japan) with the reaction program: The program as follows: denaturation (95°C for 5min), amplification and quantification (40 cycles of 95°C for 15 s and 60°C for 1 min), melting curve analysis (60–95°C, with a heating rate of 0.3°C/s). The comparative delta cycle threshold (DDCT) method was adopted to calculate relative transcript levels of 14-3-3s (Wang et al., 2017). Statistical analysis was performed by one-way analysis of variance (ANOVA) test using SPSS 19.0 software (http://www.spss.com.cn/ ). Each qPCR assay was established with three technical replicates.

### Determination of subcellular localization of potato 14-3-3s

The stop codon-free cDNA sequences of potato *14-3-3s* were inserted into pCAMBIA1305-GFP vector by homologous recombination method, which were transformed into *Agrobacterium tumefaciens GV3101*. The *Agrobacterium tumefaciens* containing fusion-expression and empty vectors infected one-month-old tobacco (*Nicotiana benthamiana*) seedlings leaves, respectively. StABI5, a reported potato *bZIP* transcription factor localized in the nucleus in tobacco, was also transformed as a control (Zhu et al., 2020). 48 h later, infected leaves were sampled for fluorescence observation under a Zeiss LSM7800 (Zeiss, Germany) confocal microscope. The DNA dye 4, 6-diamidino-2-phenylindole (DAPI) was applied to visualize the nucleus localization. The primers used in this experiment are listed in **Table S3**.

### Bimolecular fluorescence complementation assay

Full-length coding sequences without termination codon of *StABI5* and 11 potato *14-3-3*s were respectively cloned into the pUC-SPYNE and pUC-SPYCE vectors for protein-protein interaction assays, respectively. The fusion plasmids *StABI5*-cYFP and *14-3-3s*-nYFP were transformed jointly into tobacco leaves through *Agrobacterium tumefaciens* (strain GV3101) mediated methods. Then the next steps follow the subcellular localization approach described above. The primers used in this experiment are listed in **Table S3**.

## Results

### Genome-wide identification and characterization of 14-3-3s in potato

The downloaded Hidden Markov Model sequence (PF00244.20) and reported *14-3-3s* of *Arabidopsis* and tomato were served as two queries to search against potato genome database to identify the putative potato *14-3-3s*. A total of twelve nonredundant sequences were eventually retained, which were verified to contain the complete domain of 14-3-3 gene family. Refer to the naming methods in previous studies (Wang et al., 2017; Shao et al., 2021), these sequences were named as *StGF14a* to *StGF14l* based on their order in potato chromosomes (**Table S1**; **Figure S1**). The relevant information for all *14-3-3s* was collected and listed in **Table S1**. Twelve 14-3-3 members located on 7 of the 12 chromosomes and contained 249 to 285 amino acid residues with molecular weight (MW) of 28.2-32.2 kDa (**Table S1**; **Figure S1**). The predicted isoelectric point (pI) of these proteins were between 4.64 and 4.96, which was consistent with reported values of the 14-3-3 family members in other plants.

### Phylogenetic analysis of 14-3-3s in potato and other plants

**Figure 1:**
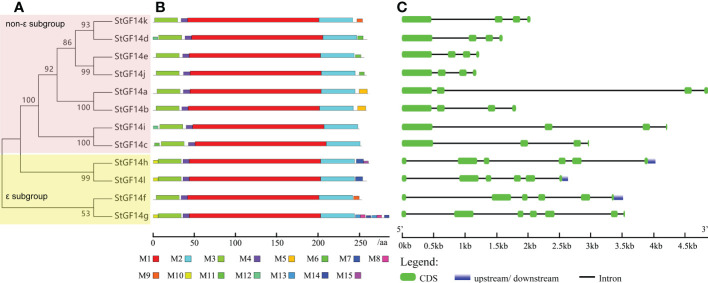
Phylogenetic relationships, conserved motifs distribution and gene structure of potato 14-3-3s. **(A)** Phylogenetic tree of twelve potato 14-3-3 proteins. **(B)** Distribution of conserved motifs within potato 14-3-3 protein sequences. The differently coloured boxes represent fifteen different conserved motifs. **(C)** Exon/intron structures of potato *14-3-3s*. The green boxes represent exons, the black lines represent introns, and the blue boxes indicates untranslated regions, and the lengths of exons can be inferred from the scale at the bottom.

To gain insight into the evolutionary relationships among members of the potato *14-3-3* family, we constructed an unrooted phylogenetic tree using maximum likelihood method. It was shown that potato 14-3-3s exhibited close phylogenetic relationships with high bootstrap values (>53%) support (**Figure 1A**). Like the classification scheme of 14-3-3 families in other plant species, potato 14-3-3 members were also able to be divided into two subgroups (ϵ subgroup and non-ϵ subgroup) based on their phylogenetic relationships. The ϵ subgroup simply contains four members, *StGF14f*, -*14h*, *-14g*, -*14l*, nevertheless, the non-ϵ subgroup contains the rest eight members, *StGF14a*, *-14b*, *-14c*, *-14d*, *-14e*, *-14i*, *-14j* and*-14k* (**Figure 1A**).

**Figure 2:**
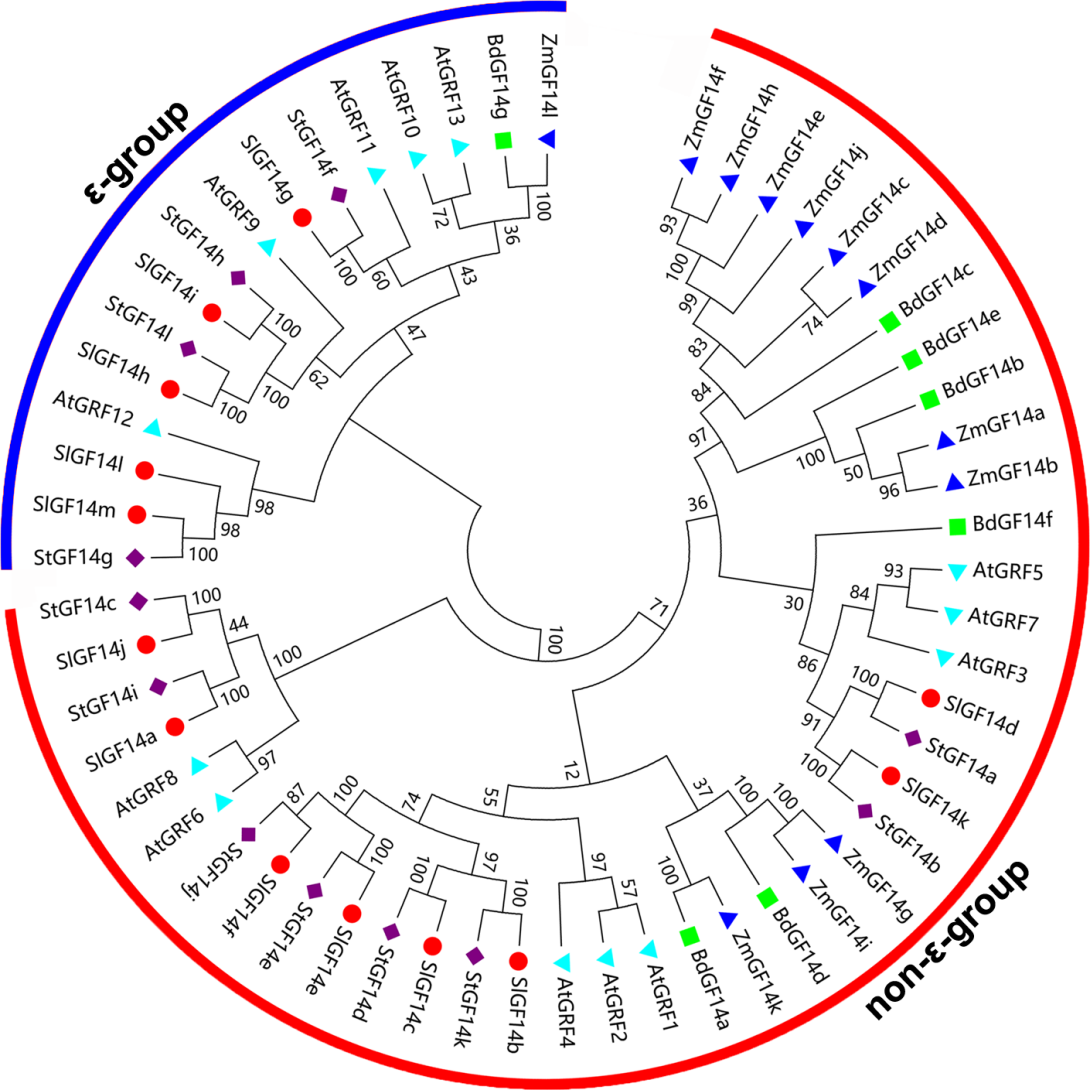
Phylogenetic analysis of 14-3-3s from five species. The phylogenetic tree ia constructed by MEGA 7.0 software using 57 14-3-3 protein sequences from *Arabidopsis* (*At*), tomato (*Sl*), maize (*Zm*), *Brachypodium distachyon* (*Bd*) and potato (*St*). The rootless tree is divided into two subfamilies, the ϵ-group is marked with blue line, and the non-ϵ-group is marked by red line.

In order to understand the relationship of 14-3-3 family members among different plant species, a total of 57 sequences from maize, *Arabidopsis*, tomato, *Brachypodium distachyon* and potato were extracted to construct an unrooted phylogenetic tree. Phylogenetic analysis showed that 14-3-3s also clustered into two groups (ϵ-group and non-ϵ-group) (**Figure 2**), the ϵ-group contains 16 members, while the non-ϵ-group contains 41 members, which was consistent with the clustering characteristics of 14-3-3s in potato (4 belong to ϵ-group and 8 belong to non-ϵ-group) and other plant species. In addition, the 14-3-3 members of potato were more closely related to these of tomato according to the phylogenetic tree. For instance, most tomato and potato 14-3-3 members were clustered together in pairs at the terminal branch of the phylogenetic tree with strong bootstrap support (**Figure 2**), which was in accordance with the evolutionary relationships among these five species.

### Gene structure and motif composition analysis of potato 14-3-3s

**Figure 3:**
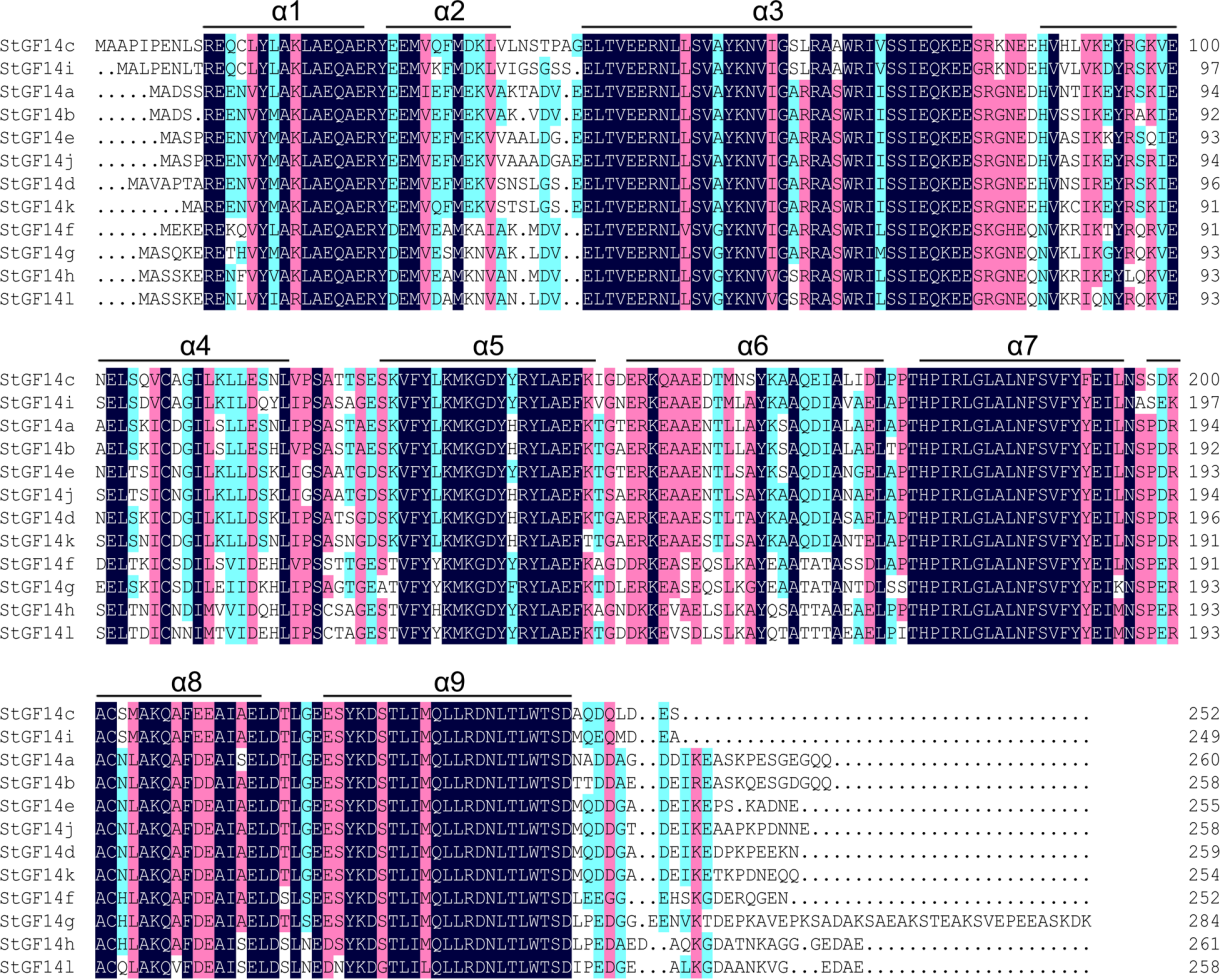
Sequences alignment of candidate potato 14-3-3 proteins. Amino acid sequences alignment of the twelve potato 14-3-3 proteins is performed using DNAMAN software. Nine α-helices were marked as α1-α9, respectively.

Gene organization analysis showed that the number of introns of potato*14-3-3s* was distributed between 3 to 6, and members in the same subgroup possessed the same number of introns as well as similar arrangement patterns of introns and exons (**Figure 1C**). For example, non-ϵ subgroup members contain three introns, while ϵ subgroup contains five introns with the exception of *StGF14g* having six introns. Although intron/exon distributions were similar among potato *14-3-3s* in the same subgroup, the intron length of each gene varies considerably, which may be one of the reasons for the functional differentiation of the same subgroup 14-3-3s (**Figure 1C**). To understand the diversity in motif composition of potato 14-3-3s, amino acid sequences were submitted to the MEME database for analysis. A total of 15 conservative motifs were identified and named as motif 1to motif 15 (**Figure 1B**; **Table S4**). Motif 1 and 2 were annotated as typical 14-3-3 family domains in the Pfam and SMART databases, which were present in all potato 14-3-3 proteins, indicating that these proteins are all genuine14-3-3 family members. However, the remaining 12 motifs had not been documented in the two current databases to date and require further study. Motif 3 and motif 4 were present in each potato 14-3-3s, suggesting these proteins may play similar roles in some pathway (**Figure 1B**). In contrast, the distribution of additional motifs was extremely variable. For example, Motif 5 only was present only in StGF14a and StGF14b, motif 6 was present only in StGF14d, StGF14e and StGF14j, and motif 9 was only present in StGF14f, suggesting that these motifs might play important roles in gene-specific functions. In addition, StGF14g contains the largest number of motifs (11), which indicated that StGF14g might play more diversified functions in potato growth and development. Similar to gene structure analysis, we found that 14-3-3s in the same subgroups showed highly similar motif arrangement, and different subgroups exhibited certain differences (**Figure 1B**). Additionally, multiple sequences alignment analysis showed that the protein sequences of the 12 potato 14-3-3s were relatively conservative and contained nine antiparallel α-helices (α1-α9) (**Figure 3**); the amino acid sequences of α- helices regions were greatly similar, while the sequences of the N-terminal and C-terminal regions exhibit a high degree of variability. Meanwhile, the three-dimensional structure predicted analysis also indicated that each potato 14-3-3 protein contained nine antiparallel α-helices, which was consistent with the result of multiple protein sequence alignment (**Figure S2**). These results also support the credibility of phylogenetic tree classification.

### Analysis of the cis-elements of potato 14-3-3s

**Figure 4:**
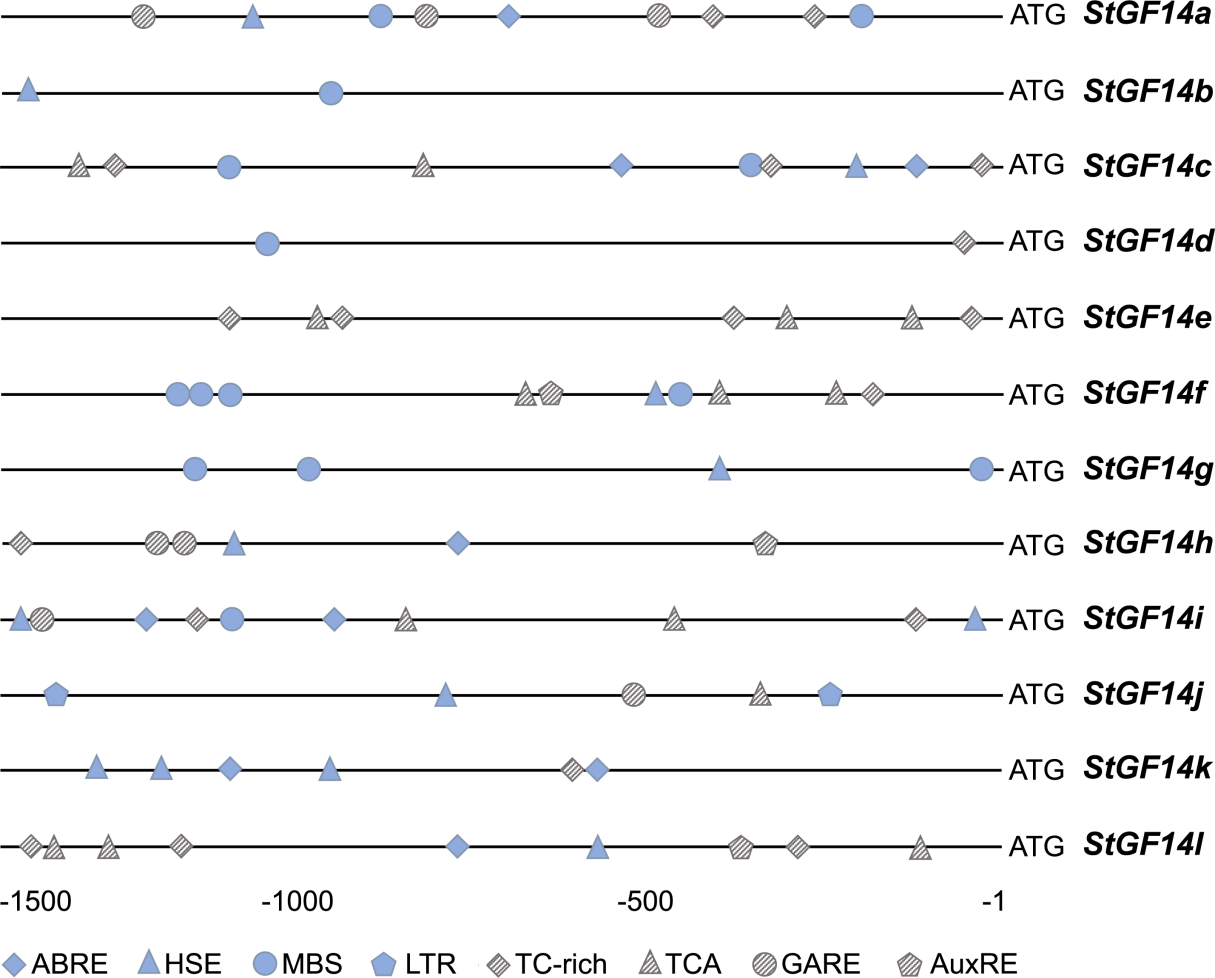
Cis-elements in the promoter regions of potato *14-3-3s*. Boxes of different colors and shapes represent eight types of cis-elements. The position of each cis-element in the promoters referring to the ruler at the bottom. ABRE (ABA responsive element), GARE (gibberellin response elements), AuxRE (auxin response element), HSE (heat stress element), MBS (drought-inducible response), LTR (low temperature response), TC-rich (defense and stress response), TCA-element (salicylic acid-related element).

It has been reported that the cis-acting elements play an influential role for gene function and regulatory patterns. The plant *14-3-3s* have been demonstrated to participate in various abiotic stress and hormonal regulation pathways. To explore the potential function of potato *14-3-3s*, the 1,500 bp upstream genomic sequence from start codon of each gene were submitted to the PlantCARE online tool for cis-acting regulatory element analysis. Eight types of *cis*-elements were detected in current study, including three hormone-related responsive elements: ABRE (ABA responsive element), GARE (gibberellin response elements), AuxRE (auxin response element), and five stress responses elements: HSE (heat stress element), MBS (drought-inducible response), LTR (low temperature response), TC-rich (defense and stress response), TCA-element (salicylic acid-related element) (**Figure 4**).

The distribution of these cis-elements in the promoter region of *14-3-3s* exhibited considerable variation, e.g., the number and types of cis-elements varied from 2 to 10 and 2 to 6, respectively. The *StGF14i* contained the maximum number (10) and types (6) of cis-elements, on the contrary, the *StGF14b* and *StGF14d* had Minimum number (2) and types (2). In addition, the HSE element was found in almost all gene promoter regions except for *StGF14d* and *StGF14e*, followed by TC-rich element in nine gene promoters (*StGF14a, -4c, -14d, -14e, -14f, -14h, -14i, -14k, -14l*), while the LTR element was only existed in *StGF14j*. These results suggest that potato *14-3-3s* are presumably regulated by multiple hormones and environmental stresses, and that different individuals may be involved in distinct signaling pathways.

### Expression profiles of potato 14-3-3s in different tissues

**Figure 5:**
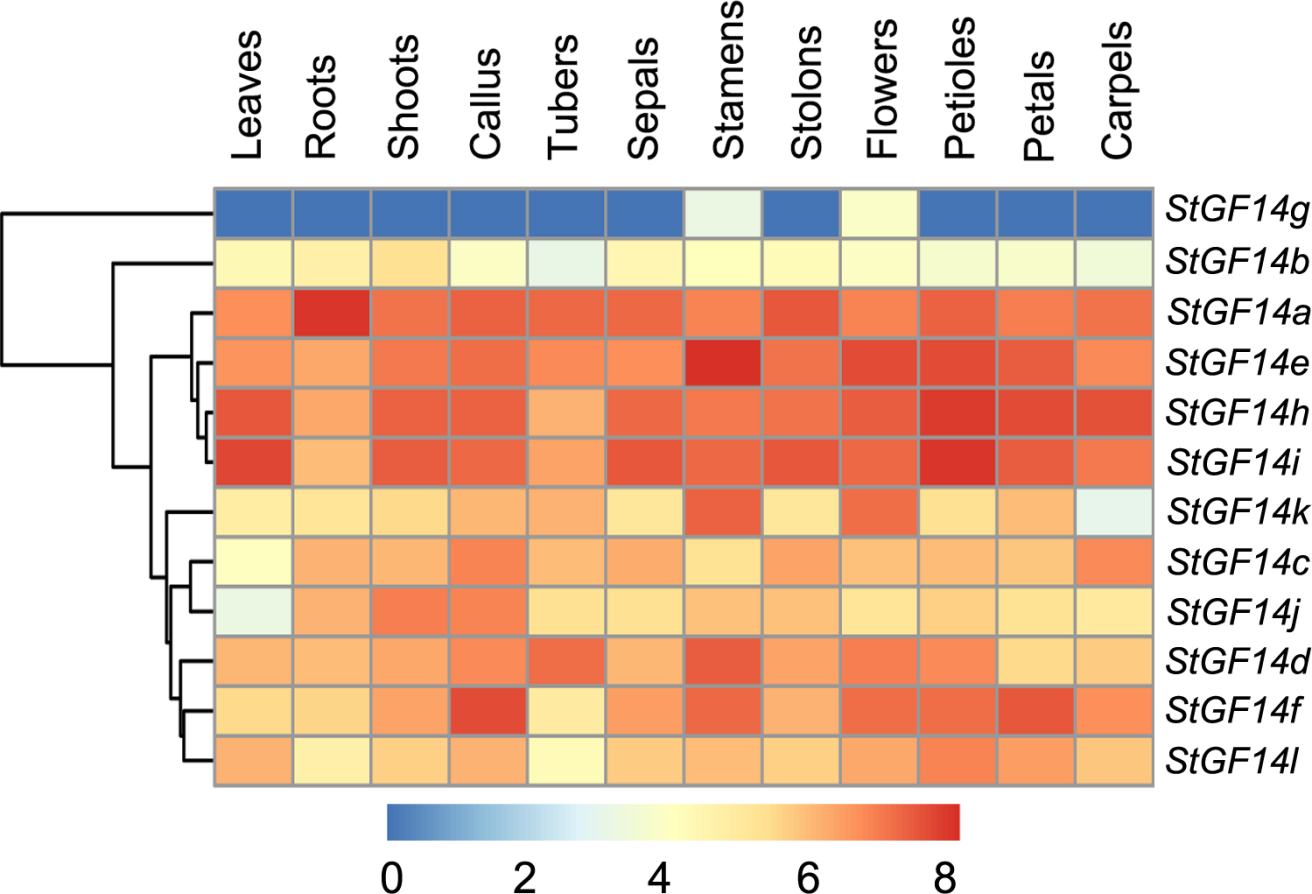
Expression profiles of potato *14-3-3s* across different tissues. Blue and red indicate low and high levels of transcript of tewlve potato *14-3-3s*, respectively. Tissues from different developmental stages are shown at the bottom of the heatmap.

To explore the temporal and spatial expression patterns of *14-3-3s* in different development stages of potato, the publicly-available transcriptome data was adopted to detect the expression level of each potato *14-3-3s* in 12 different tissues, including leaf, shoot, root, callus, tuber, sepal, stamen, stolon, flower, petiole and carpel (**Figure 5**; **Table S5**). As shown in the heatmap, different colors represent the transcript levels of *14-3-3s* in different tissues, which indicated that all genes were expressed in at least two tissues, but the expression patterns of different genes showed some degree of difference. For example, eleven *14-3-3s* (*StGF14a*, *-14b*, *-14c*, *-14d*, *-14e*, *-14f*, *-14h*, *-14i*, *-14j*, *-14k*, *-14l*) were consistently expressed in examined potato tissues, which suggested that these genes played roles in all stages of potato growth and development. On the contrary, the expression of *StGF14g* was only detected in flower and stamen at relatively low level, implying *StGF14g* may be essential for the development of flowering (**Figure 5**). In addition, we also observed that the expression level of *14-3-3s* was various in different tissues. For instance, the *StGF14a* had the highest expression level in root, *StGF14e* in stamen, *StGF14f* in callus, *StGF14h* and *StGF14i* in petiole, suggesting that these genes may play a vital role in certain specific tissues or developmental stages of potato.

### Expression profiles of potato 14-3-3s in response to drought, salt and ABA stress

**Figure 6:**
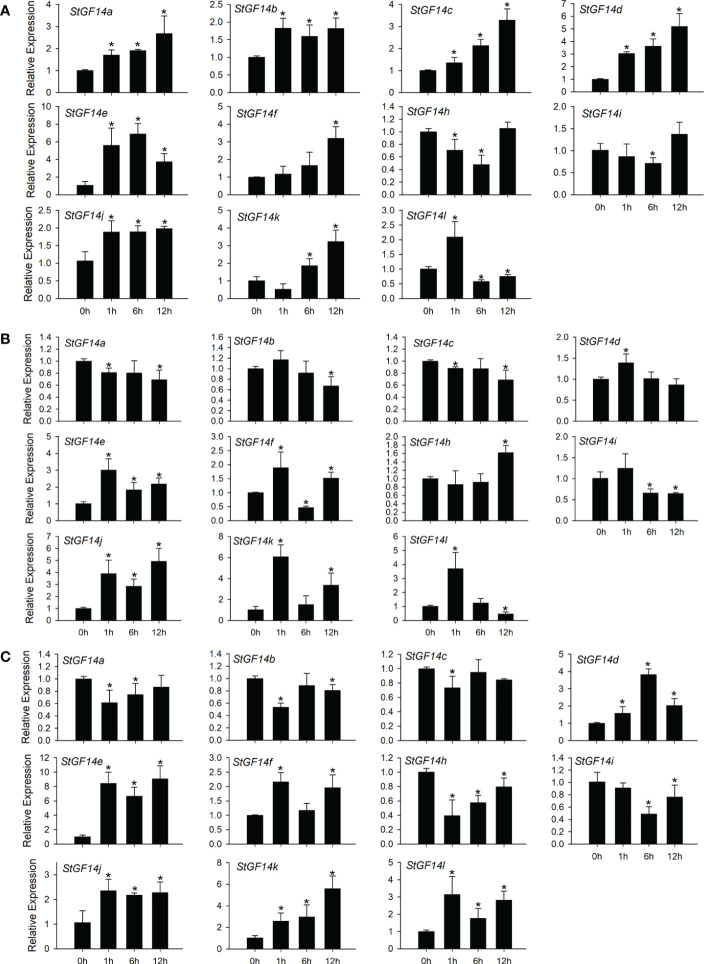
Expression analysis of potato14-3-3s in response to drought, salt and ABA treatments. The potato seedlings were treated with 100 µM ABA **(A)**, 100 mM NaCl **(B)**, and 15% PEG 6000 **(C)**. The significant differences between data were calculated using Student’s t test, and indicated with an asterisks (*), *P < 0.05.*.

The 14-3-3 family genes have been reported to be involved in multiple abiotic stress response in numerous plant species (Chen et al., 2006; Li et al., 2021; Shao et al., 2021). Previous phylogenetic analysis showed that 14-3-3 gene family were relatively conserved among potato, rice, maize and *Arabidopsis*, meanwhile, cis-element analysis indicated potato 14-3-3 family members containing abundant abiotic stress related cis-elements (**Figures 2**, **4**), which implied that potato 14-3-3 family members might also participate in abiotic stress response. Then we examined the transcriptional level of 12 potato 14-3-3 genes under drought (PEG), salt (NaCl) and exogenous ABA treatment. The results showed that almost all tested genes were up-regulated or down-regulated to some extent, except for *StGF14g*, whose expression was not detected during the whole treatment stage under three treatments (**Figure 6**), which was consistent with tissue expression patterns and further demonstrated the *StGF14g* gene only functioning during the reproductive stage of flower development. Eight genes were up-regulated under ABA treatment, including *StGF14a*, *-14b*, *-14c*, *-14d*, *-14e*, *-14f*, *-14j* and *-14k*, of which *StGF14d* and *StGF14e* displayed great amplitude of variation of expression level (>5-fold), while the *StGF14i* and *StGF14h* were slightly down-regulated (**Figure 6A**). In addition, the *StGF14l* was up-regulated at 1 h and then down-regulated at 6 h to 12 h. Under salt stress, four genes *StGF14a*, *-14b*, *-14c* and*-14i* were slightly down-regulated, while *StGF14d*, *-14e*, *-14h*, *-14j* and *-14k* were up-regulated to varying degrees (**Figure 6B**). Overall, *StGF14f* was up-regulated under salt stress treatment, but down-regulated at 6 h, whereas *StGF14l* was up-regulated at 1 h and then showed a downward trend (**Figure 6B**). Six genes, *StGF14d*, *-14e*, *-14f*, *-14j*, *-14K* and *-14l* were detected significantly up-regulated under drought treatment, by contrast, *StGF14a*, *-14b*, *-14c*, *-14h* and *-14i* were down-regulated (**Figure 6C**). It was worth noting that among the twelve genes, ten were inducible simultaneously by three types of treatment, and four genes, *StGF14e*, *-14j*, *-14k* and *-14l*, were dramatically up-regulated (**Figure 6**), which suggested that these *14-3-3s* might be involved in drought and salt stress through ABA-mediated signaling pathway.

### Subcellular localization of potato 14-3-3 proteins

**Figure 7:**
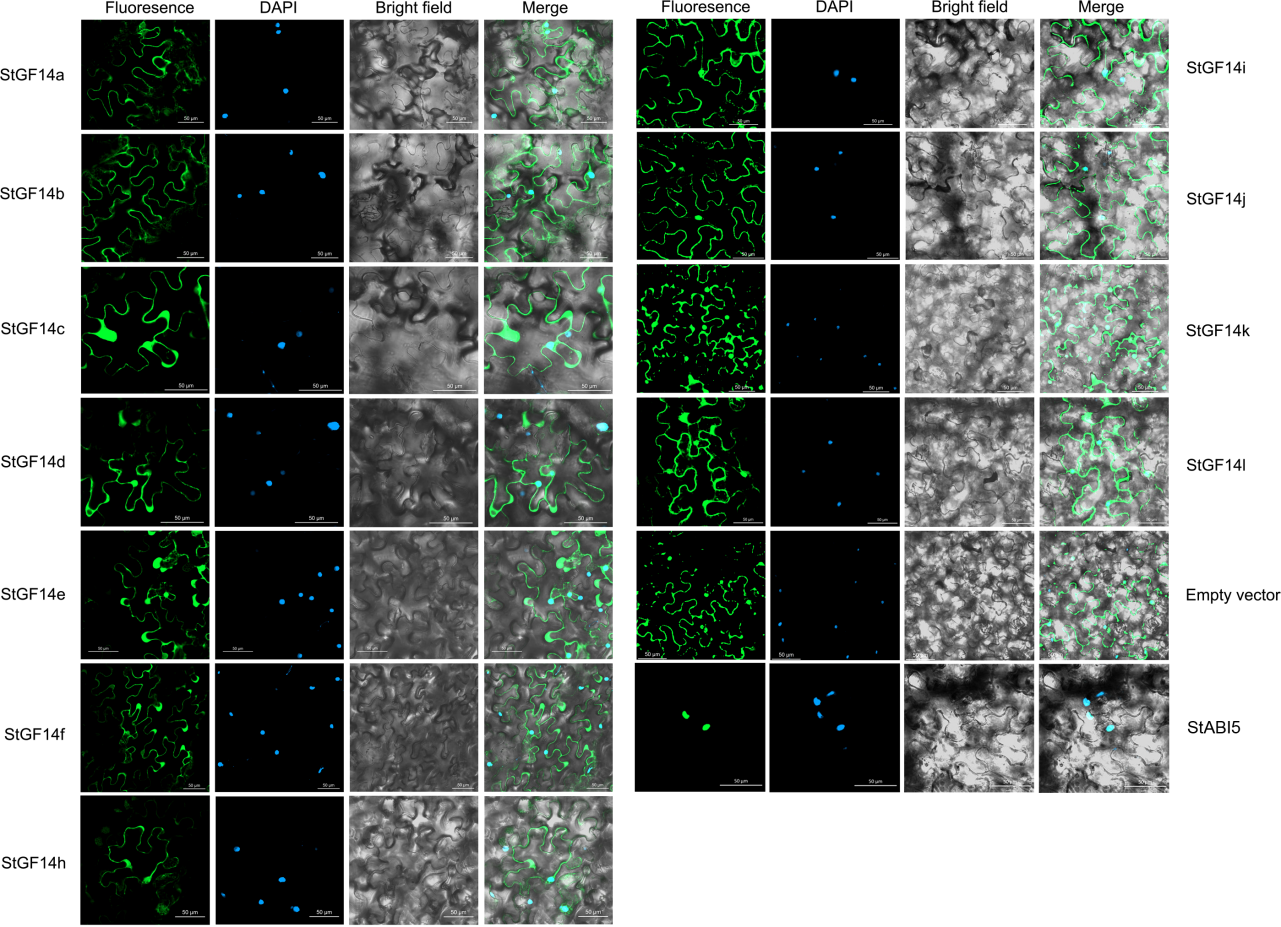
Subcellular localization analysis of potato 14-3-3s. Fusion vectors of each potato *14-3-3s* and *StABI5* are transiently expressed under control of the CaMV35S promoter in tobacco leaves and observed under a laser scanning confocal microscope. The green reprents the GFP signal, and blue represents the nucleus are stained by dapi Bars = 50 μm.

Previous studies have demonstrated that plant 14-3-3 proteins were mostly localized in both the nucleus and cytoplasm, which might be related to their extensive involvement in different biological pathway (Chen et al., 2006; Li and Sangeeta, 2010). To explore the subcellular localization of 14-3-3s in potato, the coding sequences of eleven *14-3-3s* without stop codons were cloned. On account of *StGF14g* had no expression in many potato tissues, its coding sequence was not available (**Figure 5**). Then the fusion vectors were successfully constructed through linked coding sequences of potato 14-3-3s with GFP reporter gene driven by CaMV 35S promoter, which were transiently expressed in tobacco epidermal cells to observe fluorescent signals. *StABI5*, a reported potato *bZIP* transcription factor localized in the nucleus in tobacco, was also transformed as a control. According to the result, the GFP signal of empty vector control was observed in both the nucleus and cytoplasm, and the GFP signal of *StABI5* was only present in the nucleus (**Figure 7**). The GFP signal accumulation of 11 potato14-3-3s were the same as that of empty vector control, which implicated these proteins were also located in the nucleus and cytoplasm, consistent with reported results (Zuo et al., 2021).

### Potato 14-3-3 proteins can interact with StABI5

**Figure 8:**
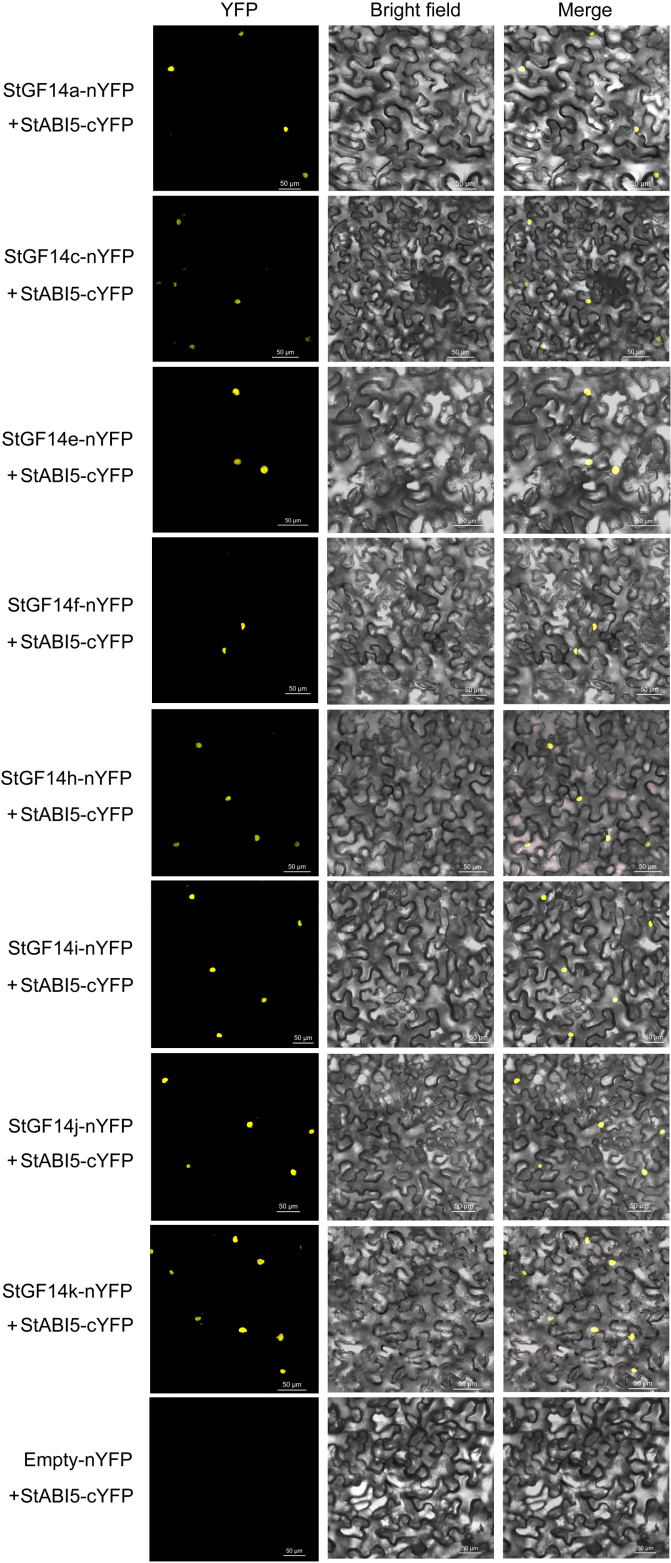
BiFC assay verifies the interactions between candidate potato 14-3-3s and StABI5. The potato 14-3-3s-nYFP and StABI5-cYFP vectors are transient transformed in tobacco leaves determined. Yellow fluorescent protein (YFP) images are detected in nulceus of tobacco leaf epidermal cells, indicating the interaction between potato 14-3-3s and StABI5. Bars = 50 μm.

Plant 14-3-3 proteins have been certified to interact with transcription factors of ABA-dependent signaling pathway to regulate the expression of downstream genes, thus participating in the regulation of plant growth and development and abiotic stress response (Casaretto and Ho, 2003). In a separate study, we identified a potato *StABI5* gene that functional analysis suggests plays a negative regulatory role in drought stress response through the ABA-dependent pathway. Therefore, a BIFC assay was performed to verify whether potato 14-3-3 protein could interact with StABI5. Our result indicated that eight 14-3-3s (StGF14a, -14c, -14e, -14f, -14h, -14i, -14j and -14k) were capable of binding to StABI5 and localized in the nucleus, while the rest three proteins could not interact with StABI5 (**Figure 8**). These results suggested that potato14-3-3s might also participate in ABA-dependent signaling pathway by interacting with transcription factors.

## Discussion

As global temperatures continue to rise, the frequency and extent of subsequent droughts will continue to increase. Mining influential functional genes and cultivating new crop varieties is one of the most effective means to improvie plant environmental adaptability. The 14-3-3s have been demonstrated to play crucial roles in response to biotic and abiotic stress (Oecking and Jaspert, 2009; Denison et al., 2011). However, compared to wheat (Shao et al., 2021), Citrus sinensis (Lyu et al., 2021), rice (Chen et al., 2006), apple (Zuo et al., 2021) and common bean (Ruihua et al., 2015), the information on potato 14-3-3s is still limited. Therefore, we performed a genome-wide identification and comprehensive analysis of the potato 14-3-3 gene family, which will provide a reference for future functional studies of potato 14-3-3s.Twelve potato *14-3-3s* from the whole potato genome were identified, which were further divided into two subgroups: ϵ group and non-ϵ group, according to their phylogenetic relationship (**Figure 1** and **Table S1**). Previous research had shown that plant 14-3-3 gene families were relatively conservative during the evolution, and they shared some similar characteristics, such as containing two subgroups, more members in non-ϵ group than in ϵ group, containing nine antiparallel α-helices, etc. (Dong et al., 1995; Yang et al., 2006). Further comprehensive analysis of potato 14-3-3 family members, we also found the more members in non-ϵ group (8) than in ϵ group (4) (**Figures 1**, **2**). On the contrary, the number of introns and extrons of ϵ group members was more than that of non-ϵ group members, which was consistent with 14-3-3 families in apple and mango, suggesting that evolution had driven this diversity and stability (Zuo et al., 2021; Xia et al., 2022). In addition, multiple sequence alignment and three-dimensional structure predicted analysis of potato 14-3-3s demonstrated that each member contained nine relatively conservative antiparallel α-helices (**Figures 3**, **S2**), further suggesting the conservative evolutionary relationship of 14-3-3 family members within and among species. However, the sequences in the N-terminal and C-terminal regions of each 14-3-3s showed a large degree of variability. This characteristic was also reflected from motif analysis where N-terminal and C-terminal motifs were extremely variable, which was regarded as the key factor for *14-3-3s* to bind various proteins and participate in different biological pathways (Coblitz et al., 2006). Potato and tomato belong to the *solanaceae* family, which diverged from common ancestry at around 7.3 Ma (Sato et al., 2012). Multispecies phylogenetic analysis showed that most potato and tomato *14-3-3*s were clustered together in pairs at the terminal branch of the phylogenetic tree with strong bootstrap support (**Figure 2**), which indicated the closer relationship between potato and tomato, and consisted with the evolutionary history (Tang et al., 2022). Taken together, these results demonstrate that potato *14-3-3s* share common properties with the plant *14-3-3* gene family, and provide further evidence for the conservation of the *14-3-3* gene family in plants.

The involvement of *14-3-3s* in a wide range of biological processes in plants is closely related to their specific expression patterns in different tissues throughout the plant growth cycle (Wilson et al., 2016). Our data displayed that potato *14-3-3s* were expressed in every tested tissue with the exception of *StGF14g* (**Figure 5**). In particular, *StGF14a*, *-14e*, *-14h* and *-14i* were consistently and strongly expressed, suggesting that these four genes played a critical role throughout growth and development in potato. However, the transcription of *StGF14g* was detected only in flower and stamen, implying that *StGF14g* may function during the reproductive phase of potato development. The involvement of plant *14-3-3s* in the regulation of flowering has been thoroughly demonstrated. For example, *Arabidopsis 14-3-3-ω*, as a bridge linker, formed a complex with the zinc finger transcription factor OXS2 (oxidative stress 2) and FT (flower-forming factor), which changed the subcellular location of FT, thereby affecting the flowering period of *Arabidopsis* (Liang and Ow, 2019). In addition, compared with wild plants, knockout of 14-3-3ν and 14-3-3μ in *Arabidopsis* also resulted in a delayed flowering phenotype (Mayfield et al., 2007). *OsGF14c*, a 14-3-3 gene in rice, has been shown to act as a negative regulator of flowering. Overexpression of *OsGF14c* resulted in a delayed flowering phenotype, while knockout it resulted in an early flowering phenotype (Asih et al., 2009). The transcript of all potato *14-3-3s* were detected in flower and stamen, indicating that these genes were also essential for potato flower development (**Figure 5**).

Increasing evidence shows that *14-3-3s* are widely involved in the signaling pathways of abiotic stress response in a variety of plants. *TFT4*, a tomato 14-3-3 gene, was significantly upregulated under salt or alkali stress (Xu and Shi, 2006). Further functional mechanism analysis indicated that *TFT4* was involved in the regulation of PKS5-J3 signaling pathway and effectively regulated the concentration of H+ in cells, thereby alleviating alkali stress (Xu et al., 2013). Similarly, the *14-3-3* gene *OsGF14e* in rice was phosphorylated by *OsCPK21* under salt stress, which promoted ABA signal transduction, thus enhancing plant salt tolerance (Chen et al., 2017). GsGF14o was demonstrated to play a negative regulatory role in drought tolerance (Sun et al., 2013). Overexpression of GsGF14o in Arabidopsis significantly reduced drought tolerance during seed germination and seedling growth with lower stomata opening, incomplete root hair development, reduced transpiration rate and water intake. In addition, recent studies have shown that the transcription of 14-3-3 family members of bananas and *Brachypodium distachyon* were regulated by a variety of abiotic stress (Cao et al., 2016; Li et al., 2016). In this study, the expression analysis of potato *14-3-3s* under drought and salt stress implied that the transcript levels of *14-3-3* family members were up- or down-regulated to varying degrees, with the exception of *StGF14g* that was not expressed (**Figure 6**). The majority of these 12 genes respond to both types of stress treatments, especially four of them, *StGF14e*, *-14j*, *-14k* and *-14l*, were significantly up-regulated. Additionally, four abiotic stress-responsive elements, HSE, MBS, TC-rich and LTR, were widely present in promoter regions of potato *14-3-3*s (**Figure 4**), suggesting the potato *14-3-3s* were potential candidates for stress-inducible gene under suitable environmental conditions. These results provide strong evidence for the role of *14-3-3s* in response to abiotic stress.

Plant hormones, such as auxin, gibberellin, abscisic acid and ethylene, play essential roles in all stages of plant growth and development and adaptation to biotic and abiotic stresses (Verma et al., 2016). The *14-3-3s* are considered to be key components of plant hormones signaling pathway, which participates in the regulation of signaling pathway by binding to relevant functional proteins. For example, the tobacco 14-3-3 proteins could bind with phosphorylated RSG (REPRESSION OF SHOOT GROWTH) and alter its subcellular localization, so that RSG could be retained in the cytoplasm instead of entering the nucleus to regulate the expression of GA biosynthesis related genes, which controlled the GA contents (Ishida et al., 2004). Similarly, 14-3-3 proteins in rice and *Arabidopsis* were capable of regulating the activity of ACS (1-aminocyclopropane carboxylic acid synthase) by binding to the phosphorylated C-terminus of ACS, thereby protecting ACS from degradation during ethylene biosynthesis (Yao et al., 2007). In addition, a recent study showed that the expression of 14-3-3 genes in citrus were induced under IAA, SA and ABA treatments (Lyu et al., 2021). In the current study, three cis-elements related to abscisic acid, gibberellin and AuxRE response were identified from the promoter regions of twelve potato *14-3-3s* (**Figure 4**). Among them, ABRE element existed in 6 *14-3-3* members’ promoters (*StGF14a*, *-14c*, *-4h*, *-14i*, *-14k*, *-14l*), GARE element existed in 4 *(StGF14a*, *-14h*, *-14i*, *-14j*), and AuxRe exists in 3 (*StGF14f*, *-14h*, *-14l*), respectively, suggesting that potato 14-3-3 members may also involve in hormone signaling pathways.

Abscisic acid (ABA), an essential plant hormone, plays a critical regulatory role in plant response to various adverse environmental stimuli (Raghavendra et al., 2010). Under drought stress, ABA content is rapidly accumulated in plant, which activates or inhibits the expression of stress-related genes in ABA- dependent signaling pathway to improve plant stress tolerance through regulating stomatal closure to reduce transpiration rate, regulating cell osmotic pressure, and changing root morphology to obtain additional soil water (Zhang et al., 2006). The *14-3-3s* have been shown to be crucial members of ABA-dependent signaling pathways, which modulates the expression of downstream ABA-responsive genes by interacting with transcription factor genes in plants. Under the exogenous ABA treatment, two potato 14-3-3 genes, *StGF14h* and *StGF14i* were slightly down regulated, while other nine genes (*StGF14a*, *-14b*, *-14c*, *-14d*, -*14e*, -*14f*, -*14j*, -*14k*, -*14l*) were up regulated to varying degrees (**Figure 6**), which suggested that potato 14-3-3 genes might also participate in ABA-dependent signaling pathway.

The ABA insensitive 5 (*ABI5*) is a transcription factor with basic leucine zinc finger structure (*bZIP*), which is a key target gene in ABA-dependent signaling pathway (Miura et al., 2019). In barley, the 14-3-3 proteins acted as a linker to associate the ABA effector VP1 (viviparous 1) with HvABI5 to jointly regulate the expression of downstream ABA response genes (Casaretto and Ho, 2003). In the current study, The BIFC assay showed that eight potato 14-3-3 proteins, StGF14a, -14c, -14e, -14f, -14h, -14i, -14j and -14k could interact with StABI5 (**Figure 8**). In addition, our separate study showed that the *StABI5* played a negative regulatory role in potato drought stress response (data not shown), indicting these 8 14-3-3s might jointly participate in drought stress response through the interaction with StABI5. These results further confirm that potato 14-3-3 members participate in ABA-dependent signaling pathway through interactions with downstream TFs.

In conclusion, we performed a comprehensive analysis of potato 14-3-3s in this study. Twelve potato 14-3-3 proteins were identified and they exhibited distinct transcript accumulation in different tissues during the growth and development process as well as in response to a variety of abiotic stresses. In addition, the key target member in ABA-dependent signaling pathway StABI5 was demonstrated to act as binding partners of potato 14-3-3s. These results further certified the correlation between 14-3-3s and adversity stress response, which also provided new clues to discover important candidate genes for potato resistance breeding. Nevertheless, future studies on the functional mechanism of potato14-3-3s regulation of stress tolerance should be strengthened.

## Data availability statement

The datasets presented in this study can be found in online repositories. The names of the repository/repositories and accession number(s) can be found in the article/**Supplementary Material**.

## Author contributions

QW and HL conceived and designed the study. CY, YF, YW and YD performed the experiments. QW and PJ analyzed data. QW wrote the manuscript. All authors contributed to the article and approved the manuscript.

## Supplementary material

Supplementary Figure 1 Chromosomal locations of potato 14-3-3s.

Supplementary Figure 2 The predicted 3D models of twelve 14-3-3 proteins in potato. The α1-α9 represent nine α-helices in the protein structures, respectively, and marked with different colour curves.

Supplementary Figure 3 BiFC assay verifies the interactions between candidate potato 14-3-3s and StABI5.

Supplementary Table 1 Characteristics of 14-3-3 genes in potato.

Supplementary Table 2 The nomenclature and characteristics of *14-3-3s* in four representative species.

Supplementary Table 3 List of gene-specific primers used in the present study.

Supplementary Table 4 Sequences of conserved motifs identified in potato 14-3-3 proteins.

Supplementary Table 5 FPKM of potato *14-3-3s* in different tissues.
