# Supplementary figures and images for: Genome-Wide Identification of 14-3-3 gene family reveals their diverse responses to abiotic stress by interacting with StABI5 in Potato (Solanum tuberosum L.)
Source: Front Plant Sci. 2023 Jan 9;13:1090571. doi: 10.3389/fpls.2022.1090571 (PMC9868832; doi:10.3389/fpls.2022.1090571)

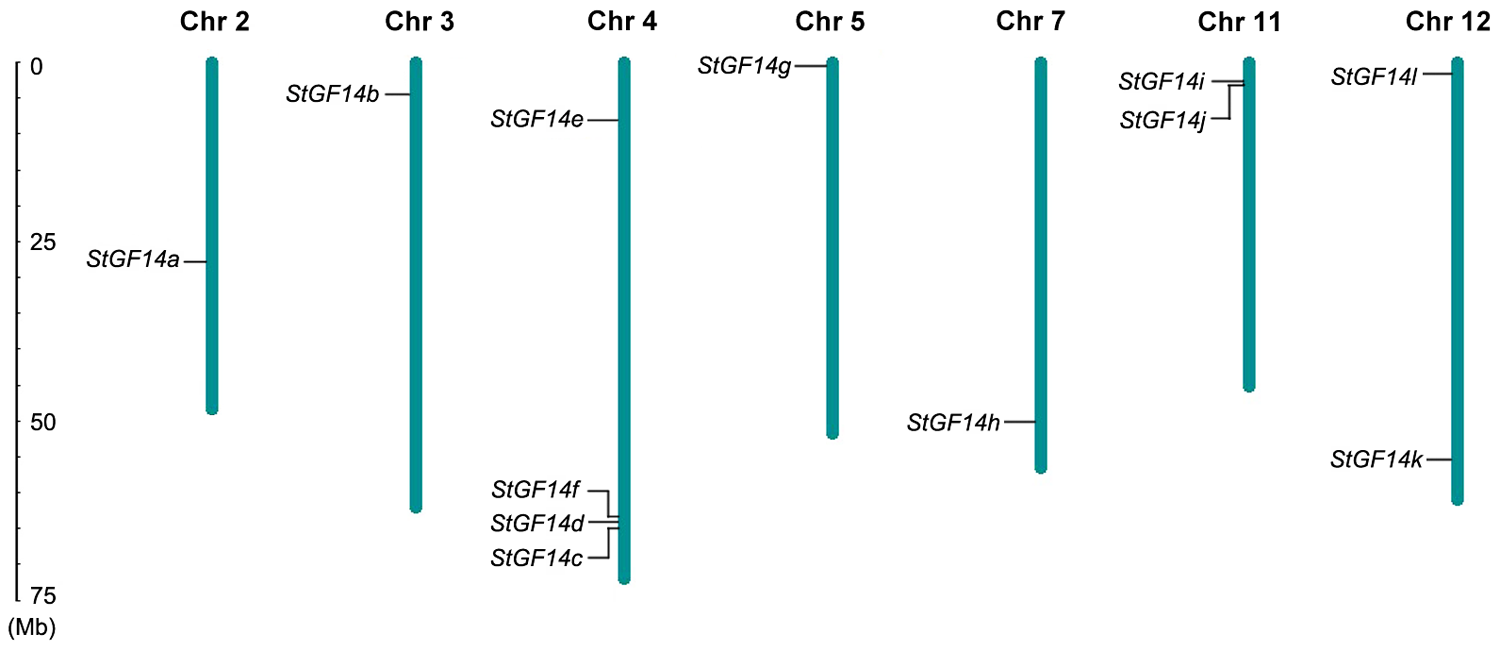

Supplement: Supplementary Figure 1 — Chromosomal locations of potato 14-3-3s. [file Image_1.tif]

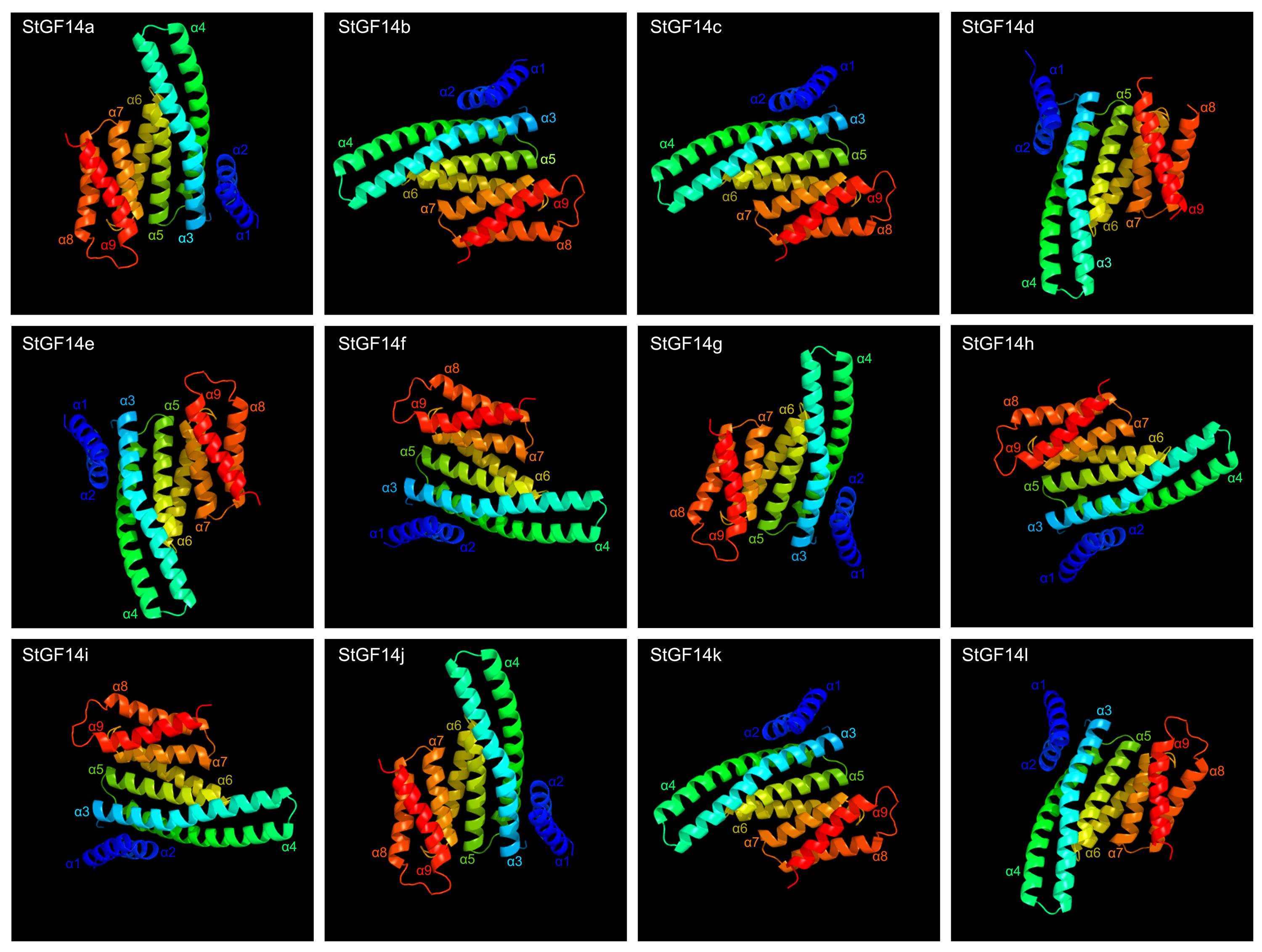

Supplement: Supplementary Figure 2 — The predicted 3D models of twelve 14-3-3 proteins in potato. The α1-α9 represent nine α-helices in the protein structures, respectively, and marked with different colour curves. [file Image_2.tif]

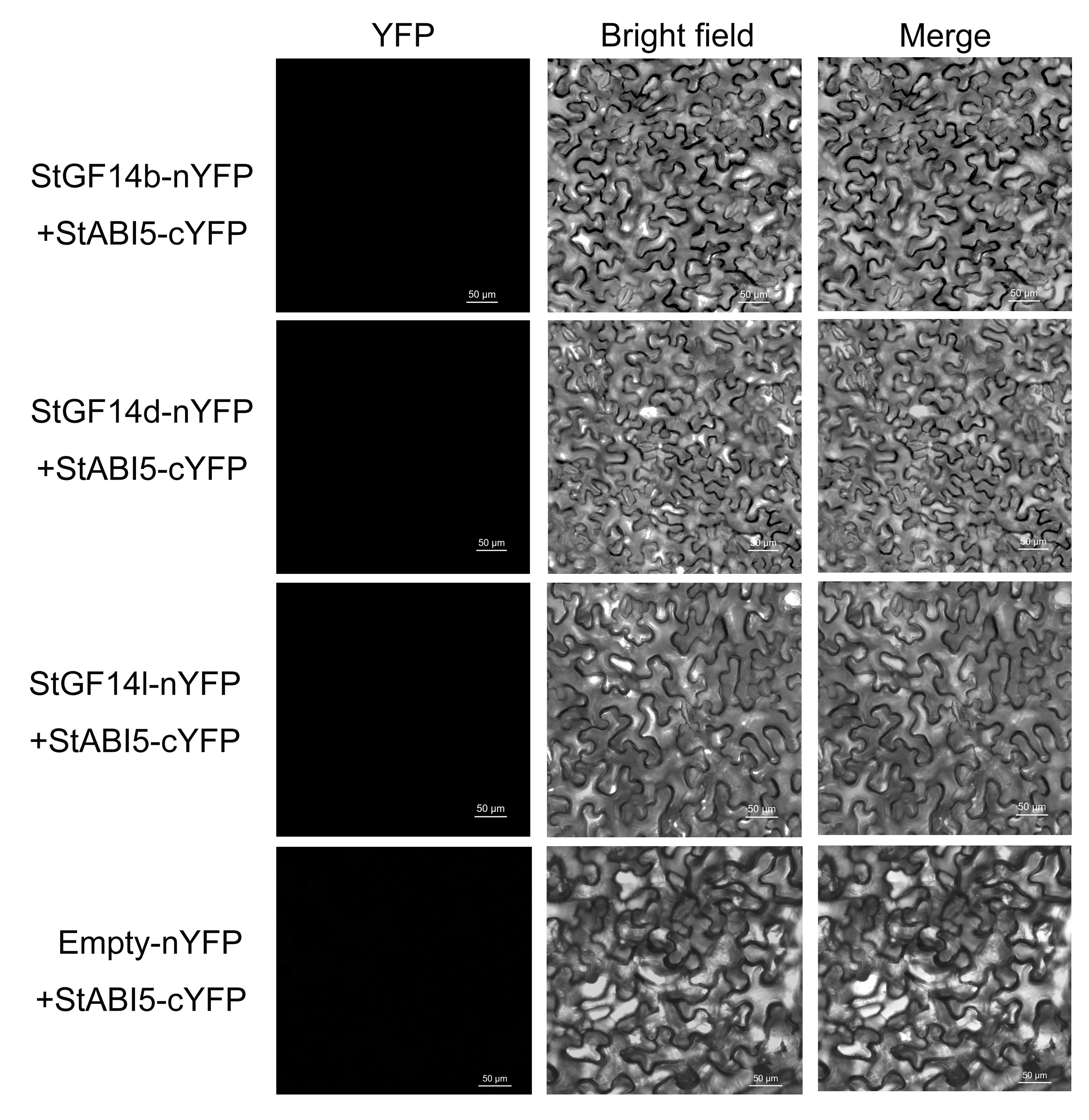

Supplement: Supplementary Figure 3 — BiFC assay verifies the interactions between candidate potato 14-3-3s and StABI5. [file Image_3.jpeg]
