# Supplementary material for: Genome-Wide Identification of 14-3-3 gene family reveals their diverse responses to abiotic stress by interacting with StABI5 in Potato (Solanum tuberosum L.)
Source: Front Plant Sci. 2023 Jan 9;13:1090571. doi: 10.3389/fpls.2022.1090571 (PMC9868832; doi:10.3389/fpls.2022.1090571)
Supplement: Supplementary Table 4 — Sequences of conserved motifs identified in potato 14-3-3 proteins. [file Table_4.docx]

| **Motif** | **Width** | **Motif sequences** | **Annotation** |
| --- | --- | --- | --- |
| 1 | 159 | RNLLSVAYKNVIGARRASWRIISSIEQKEESRGNEEHVKSIKEYRSKIESELSKICNGILKLLDSHLIPSASTGESKVFYLKMKGDYYRYLAEFKTGAERRNLLSVGYKNVIGARRASWRIISSIEQKEESRGNEQHVHCIKEYRQKVENELSKICNGILKLLDSHLIPSCTTGESKVFYLKMKGDYHRYLAEFKTGDERKEAAENTLKAYKAAQDIANAELPPTHPIRLGLALNFSVFYYEILNSPDRACHLAKQAFD | 14-3-3 |
| 2 | 41 | EAIAELDTLGEESYKDSTLIMQLLRDNLTLWTSDMQDDGED | 14-3-3 |
| 3 | 28 | REENVYMAKLAEQAERYEEMVEFMEKVA | Unknown |
| 4 | 8 | DEELTVEE | Unknown |
| 5 | 10 | SKPESGEGQQ | Unknown |
| 6 | 6 | APKPEN | Unknown |
| 7 | 9 | KGDAANKVG | Unknown |
| 8 | 6 | PKAVEP | Unknown |
| 9 | 7 | KGDEEQG | Unknown |
| 10 | 6 | MASSKE | Unknown |
| 11 | 7 | IKEDPKP | Unknown |
| 12 | 6 | MALPPN | Unknown |
| 13 | 6 | EVKSDE | Unknown |
| 14 | 6 | SKDKKS | Unknown |
| 15 | 6 | GEPDKE | Unknown |

**Table S3 Sequences of conserved motifs identified in potato 14-3-3 proteins**
